# Chinese Herbal Medicine for Aspirin Resistance: A Systematic Review of Randomized Controlled Trials

**DOI:** 10.1155/2014/890950

**Published:** 2014-02-20

**Authors:** Ai-ju Liu, Hui-qin Li, Ji-huang Li, Yuan-yuan Wang, Dong Chen, Yan Wang, Guo-qing Zheng

**Affiliations:** ^1^Department of Neurology, The Second Affiliated Hospital of Wenzhou Medical University, Wenzhou 325027, China; ^2^Department of Cardiology, The Second Affiliated Hospital of Wenzhou Medical University, Wenzhou 325027, China

## Abstract

Aspirin resistance (AR) is a prevalent phenomenon and leads to significant clinical consequences, but the current evidence for effective interventional strategy is insufficient. The objective of this systematic review is thus to assess the efficacy and safety of Chinese herbal medicine (CHM) for AR. A systematical literature search was conducted in 6 databases until December 2012 to identify randomized controlled trials (RCTs) of CHM for AR. As a result, sixteen RCTs with a total of 1011 subjects were identified, suggesting that the interests of the medical profession and the public in the use of CHM for AR have grown considerably in the recent years. Tongxinluo capsule and Danshen-based prescriptions were the most frequently used herbal prescriptions, while danshen root, milkvetch root, Leech, and Rosewood were the most frequently used single herbs. Despite the apparent reported positive findings, it is premature to determine the efficacy and safety of CHM for the treatment of AR due to poor methodological quality and insufficient safety data. However, CHMs appeared to be well tolerated in all included studies. Thus, CHM as a promising candidate is worthy of improvement and development for further clinical AR trials. Large sample-size and well-designed rigorous RCTs are needed.

## 1. Introduction

### 1.1. Description of the Condition

Cerebral infarction and myocardial infarction can be fatal and seriously affect patients' living quality, and their incidence will increase greatly in recent decades, especially in developing countries, due to more work pressure and population aging [1, 2]. Antiplatelet therapy is an important prevention measure for lowering the likelihood of myocardial infarction and stroke in high risk vascular patients [3, 4]. Among antiplatelet drugs, acetylsalicylic acid, known as aspirin, became a cornerstone in the treatment of cardio/cerebrovascular diseases and has been shown to be effective in the secondary prevention of vascular events [4, 5]. The use of aspirin can be dated back to early Hippocrates who used willow leaves, rich in Acetylsalicylic acid, to relieve the aches associated with multiple illnesses in ancient Greece [6, 7]. Reverend Edmund Stone who progressed aspirin from folk remedy to a blockbuster drug in 1763 isolated salicin, the glycoside of salicylic acid and the active ingredient of aspirin, from the bark of a willow tree in England [6, 7]. Aspirin was not born until 1897 when the Bayer company hired Felix Hoffman, a German chemist, who created acetylsalicylic acid by acetylating salicylic acid's hydroxyl ring resulting in well-tolerated compound [6, 8]. After that time, people around the world began to appreciate its fast onset and predictable relief. However, for all that has been discovered about its beneficial effects, sometimes aspirin's effects are like an infatuation, eventually, they fade or disappear [6]. That is to say, despite the impressive efficacy and safety of low dose aspirin in preventing atherothrombosis, many patients who were receiving aspirin therapy still suffered significant clinical consequences such as stroke, myocardial infarction, and other vascular death events [9]. This clinical phenomenon was called aspirin resistance (AR). The reported frequency of AR was high and variable based on the different dosage of aspirin and biochemical method testing platelet function [9]. The study by Gum et al. [10] reported a 5% prevalence of AR in patients taking aspirin for prevention of atherosclerotic events, whereas another study reported a 60% rate of AR in patients with intermittent claudication [11]. A large systematic review including 42 studies reported a mean prevalence of AR as 25% in patients taking aspirin for secondary prevention [9]. Therefore, the intervention of AR is an important measure for the prevention of cardio/cerebrovascular diseases.

Aspirin resistance has been defined by clinical and/or laboratory criteria, but a generally accepted definition based on valid diagnostic criteria has not been established. The possible first description of AR has been debated since the 1980s; FitzGerald et al. [12] demonstrated that adenosine diphosphate (ADP) induced platelet aggregation was maximally inhibited by aspirin 40~80 mg/day, but values returned to baseline with chronic administration at higher doses of up to 2,600 mg/day. Unfortunately, “aspirin resistance” was not actually used until 10 years later when Helgason et al. [13] reported inadequate inhibition of platelet aggregation by aspirin in some patients suffering from cerebrovascular disease. However, “aspirin resistance” remains yet with no standard definition. This term used in the literature mainly included the following [14–16]. (1) Clinical “aspirin resistance” or “treatment failure” is the occurrence of acute thrombotic events despite regular aspirin therapy. (2) Laboratory-defined “aspirin resistance” or aspirin nonresponsiveness is present when *in vitro* platelet reactivity is not properly blocked despite the use of aspirin, ranged from the specific failure to inhibit thromboxane A2 (TXA2), failure to inhibit a test of platelet function that is dependent on TXA2 production, and the very broad failure to inhibit one or more platelet function assays. (3) “High on-treatment residual platelet reactivity” found *in vitro* in patients with antiplatelet therapy does not necessarily mean “resistance” to the antiplatelet drug from a pharmacological point of view. (4) Some authors distinguished types of “aspirin resistance” [17, 18] as follows: (a), pharmacokinetic resistance: platelet aggregability was successfully inhibited by *in vivo* addition of aspirin; (b), pharmacodynamic resistance: platelet aggregability continued when *in vitro* aspirin was added, with the persistent formation of TXA2; (c), pseudoresistance resistance: platelet aggregability was continued even when *in vitro* aspirin was added, but there was successful inhibition of TXA2 formation.

The key laboratory parameters are used to examine platelet responses to aspirin in a laboratory study using measures such as: (a), Bleeding time: measurement of the time necessary for bleeding to stop after standardized skin incision; (b), light transmission aggregometry: analysis of light transmission after stimulation of platelets with arachidonic acid, collagen, ADP, or other agonists in anticoagulated platelet-rich plasma; (c), Impedance aggregation (whole blood aggregometry): Measurement of electrical impedance between two electrodes in whole blood after stimulation with arachidonic acid, collagen, ADP, or other agonists; (d), platelet function analyzer-100 (PFA-100), assessment of aggregation under high shear; whole blood is aspirated through an aperture coated with collagen and either epinephrine or ADP; (e), rapid platelet function assay (VerifyNow Aspirin system): analysis of light transmission in a test cartridge containing fibrinogen-coated beads in whole blood; (f), levels of serum TXB2: radioimmunoassay or ELISA; TXA2 is rapidly converted into the stable metabolite TXB2; (g), urinary 11-dehydro-TXB2: measurement using radioimmunoassay or ELISA; systemic TXB2 undergoes hepatic transformation into 11-dehydro-TXB2; (h), flow cytometry: automated laser detection of platelet activation markers (e.g., P-selectin, CD63, changes in GP IIb/IIIa complex conformation) using antibodies; (i), thromboelastography: analysis of clot strength from formation to lysis after stimulation with arachidonic acid or ADP [14, 19–21].

### 1.2. Description of the Intervention

The current evidence for effective intervention of AR is insufficient. Clinical treatment of AR such as an increase in aspirin dose and the addition of other antiplatelet drugs is usually not effective [21, 22]. Aspirin doses of 75–150 mg daily were at least as effective as higher doses daily, which was demonstrated in trials comparing different doses of aspirin versus no aspirin, and the addition of dipyridamole to aspirin did not reduce the incidence of ischemic events significantly comparing with aspirin alone [23]. Clopidogrel, an oral thienopyridine, can selectively and irreversible block the P2Y12 platelet receptor for ADP, thereby inhibiting ADP induced platelet aggregation. Aspirin plus clopidogrel can inhibit the two main pathways of platelet aggregation, the arachidonate/TXA2 and the ADP pathways [24]. The study by Dropinski et al. [25] reported that clopidogrel had significant effect on AR for patients with coronary artery disease. However, another study by Lev et al. [26] reported about 50% of the AR patients who were also resistant to the effects of clopidogrel because AR patients have a low inhibitory response to clopidogrel. Dipyridamole is a phosphodiesterase (PDE) inhibitor and has been used in antithrombotic treatment [27]. Although the addition of dipyridamole to aspirin has been reported be more effective in the secondary prevention of stroke than aspirin alone [28], there was insufficient evidence to indicate that dipyridamole was effective for AR in patients with vascular disease [29]. Cilostazol, an inhibitor of phosphodiesterase III, has antiplatelet aggregation activity by inhibiting the conversion of cyclic adenosine monophosphate (cAMP) to 5′-AMP and eventually potentiates the glycoprotein IIb-IIIa inhibitory signals to increases the level of cAMP [30, 31]. However, the current evidence only showed a tendency in supporting the additional use of cilostazol because cilostazol add-on therapy did not reduce the rate of AR [32]. Furthermore, high doses of aspirin and the addition of other antiplatelet drugs can increase the risk of gastrointestinal hemorrhage and other adverse events [22].

Faced with the limitations of the presently available treatments, complementary and/or alternative medicine (CAM) is thus increasingly being sought to treat AR worldwide. Currently, the usage of CAM has increased between the general population and medical personnel in many countries [50]. One recent study has reported that treatment of AR patients by adding one kind of CAMs, omega-3 fatty acids, can improve response to aspirin and effectively reduces platelet reactivity [51]. In China, the important characteristic of China's national medical system is that traditional Chinese medicine (TCM) and Western medicine complement and cooperate with each other, being responsible together for the health care of Chinese people [52]. In the past decades, Chinese herbal medicine (CHM), as one form of CAMs, has been widely used throughout China and elsewhere in the world for the treatment of AR [53].

### 1.3. How the Intervention Might Work

**Figure 1:**
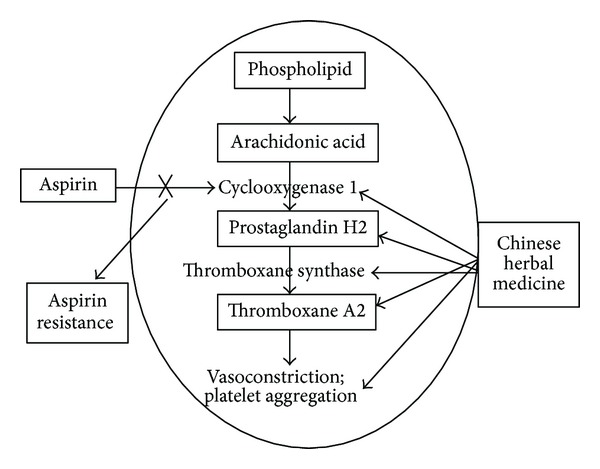
Mechanism of Chinese herbal medicine for aspirin resistance.

Pharmacological studies have found the multitarget intervention effects of CHM on the pathophysiology of AR (Figure 1). For example, Buyang Huanwu decoction (BHD), a well-known classic CHM prescription for stroke, can inhibit platelet aggregation by interfering with cyclooxygenase-1 (COX-1) expression on platelet and endotheliocyte in rabbits [42]. Some CHMs for promoting blood circulation such as *Rheum palmatum* and *Erigeron breviscapus* and some tonic herbs such as *Codonopsis pilosula*, *Astragalus membranaceus*, *Angelica sinensis*, and ginsenosides play an important role in the inhibition of TXA2 synthesis *in vitro* in the porcine lung microsoma as donor of enzymes [54]. Ginkgolide C from *Ginkgo biloba* leaves is an inhibitor of collagen stimulated platelet aggregation by reducing TXA2 formation in rat blood sample with a dose-dependent effect [55]. The ingredients of CHM flavones can suppress platelet aggregability through inhibiting TXA2 receptor [44]. Panaxtrial saponins can inhibit platelet aggregation and TXA2 releasing induced by collagen and arachidonic acid, improve vascular endothelial function, reduce platelet surface activity, inhibit blood platelet adhesion, reduce blood viscosity, improve microcirculation, and resist thrombosis formation in patients with cardiovascular disease [56, 57]. *Atractylodis lanceae* rhizoma can inhibit collagen- or TXA2-induced platelet aggregation by suppressing the collagen-induced signal pathway, which is upstream of the release of TXA2 in rabbit blood sample with a concentration-dependent effect, and Poria(Indian bread) inhibits TXA2 receptor-mediated platelet aggregation by suppressing Gq-mediated signaling pathway in rabbit blood sample with a concentration-dependent effect [58]. Ilexonin A, an active ingredient of Maodongqing, is a potent inhibitor of vWF binding and vWF mediated platelet activation and aggregation in healthy volunteer blood sample [59]. Ilexonin A can also inhibit arachidonic acid- (AA-) induced platelet aggregation by interfering with TXA2 formation in rabbit blood sample with a dose-dependent effect [60]. Danshen-based preparations have been used for hundreds of years in treatment of cardiovascular ischemic diseases [61] with the properties of inhibiting prostaglandin synthesis and platelet adhesion and aggregation in diabetic patients [62] and with suppression of both the formation and the release of TXA2 in rats [63]. Thus, CHM has the pharmacological effects of inhibiting TXA2 and consequently TXA2-dependent platelet functions.

### 1.4. Why It Is Important to Do This Review

Owing to the significant health risk of AR and the limitations of currently available conventional therapies, there have been a number of controlled studies over the past decade to evaluate the efficacy and safety of CHM for AR. However, to date, there was no systematic review available to determine the efficacy of CHM for AR. In addition, many of the current clinical trials of CHM for AR are thought of as “not so good” studies according to Cochrane criteria. In a TCM reviewing process, researchers may need to include such papers to identify current problems and areas worthy of improvement and future development [64]. Therefore, the objective of present systematic review is to assess the efficacy and safety of CHM for AR.

## 2. Methods

This systematic review is conducted according to the preferred reporting items for systematic reviews and meta- analyses: the PRISMA statement [65].

### 2.1. Eligibility Criteria

Types of studies: studies have to be randomized clinical trials (RCTs) concerning specifically the effectiveness of CHM for AR, regardless of blinding, publication status, or language. Studies of quasi-RCTs which are allocating participants by date of birth, day of the week, medical record number, and month of the year were excluded.

Types of participants: criteria for the diagnosis of AR are as follows: >70% platelet aggregation rate induced by 10 *μ*mol/L ADP and >20% aggregation rate induced by 0.5 g/L arachidonic acid (AA). The diagnostic criterion of aspirin semiresistance (ASR) is meeting one of the two conditions above. The exclusion criteria of AR are the following: (1) aspirin allergy or asthma; (2) all kinds of blood disease, hemorrhagic disease, or bleeding tendency; (3) platelet count >450 × 10^9^/L or <100 × 10^9^/L; (4) using ticlopidine, clopidogrel, dipyridamole, unfractionated heparin, low molecular heparin, and other nonsteroidal anti-inflammatory drugs during the last 2 weeks of observation period; (5) active gastric ulcer patient; (6) gout patient.

Types of interventions: any intervention using CHM monotherapy or adjunct therapy for AR in the treatment group was included regardless of the dose, frequency, and intensity of CHM. The interventions of control groups were regular low dose aspirin treatment (aspirin 100 mg/d), high dose aspirin treatment (aspirin 300 mg/d), no intervention, or other antiplatelet drug treatment such as dipyridamole, clopidogrel, and cilostazol. The treatment course was at least 2 weeks in these studies.

Types of outcome measures: the primary outcome measurement was platelet aggregation rate induced by AA and/or ADP at the end of treatment course. The secondary outcome measurement was the clinical efficacy rate and adverse events at the end of treatment course. The clinical efficacy rate was defined as the decreased value of platelet aggregation rate before and after treatment. The standard of clinical efficacy rate met any one of the following: (1) AR becomes ASR or aspirin sensitive; (2) ASR becomes aspirin sensitive; (3) the decreased value of platelet aggregation rate is more than 10% and platelet active value is normal. The diagnosis criteria of aspirin sensitive are as follows: ≤70% platelet aggregation rate induced by 10 mL ADP and ≤20% aggregation rate induced by 0.5 g/L AA.

### 2.2. Literature Search

We electronically searched CENTRAL, PubMed, EMBASE, Chinese National Knowledge Infrastructure (CNKI), VIP information database, and Wanfang Data Information Site until December 2012 to identify RCTs involving CHM for AR patients. In addition, we hand-searched a list of medical journals relevant to this topic and references from relevant articles. Using free text and the medical subjects headings (MeSH) terms combined “aspirin resistance” and “Chinese herbal medicine or traditional Chinese medicine or Chinese materia medicine or integration of Chinese herbal and Western medicine.”

### 2.3. Study Selection and Data Extraction

Two investigators independently reviewed the titles and abstracts to select potential references. Then, the two investigators read the selected articles independently and made a final decision for selection or not. Key data were extracted according to standardized criteria, including patients, methods, interventions, and outcomes. Disagreements were settled through discussion or consultation with one correspondence author.

### 2.4. Risk of Bias in Individual Studies

The methodological quality of RCTs was assessed independently by two investigators using the 12-item criteria from Cochrane Back Review Group [66]. All data were analyzed using Review Manager 5.0 software. We are reconciling any discrepancy through discussion with one correspondence author.

## 3. Results

### 3.1. Description of Studies

**Figure 2:**
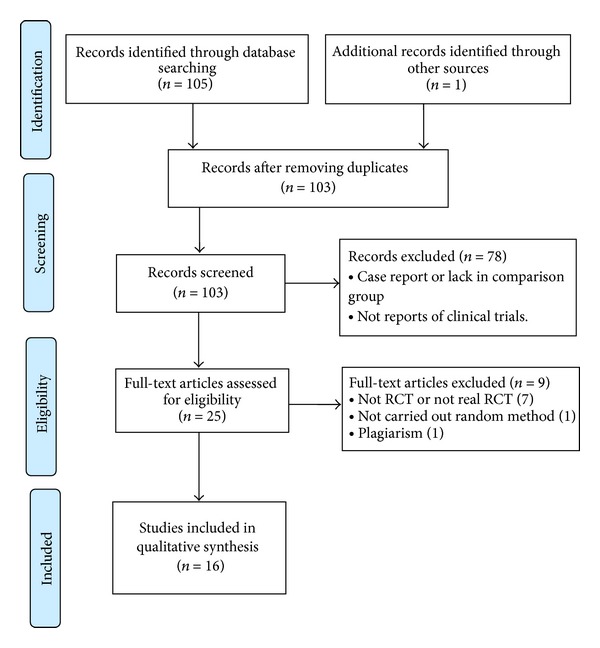
PRISMA 2009 flow diagram.

We identified 106 potentially relevant articles. After removal of duplicates, 103 records remained. After going through the titles and abstracts, 78 papers were excluded on the basis that they were nonclinical trials, case report, or lack of comparison group. By reading the full text of the remaining 25 articles, 7 were excluded for not being RCTs or not real RCTs; 1 paper was removed due to plagiarism; 1 paper was excluded as a result of not carrying out random method. Ultimately, 16 eligible studies were selected out for this systematic review. The screening process is summarized in a PRISMA 2009 flow diagram (Figure 2).

### 3.2. Characteristics of Included Studies

There were a total of 1011 subjects in the 16 included trials of CHM for AR published between 2006 and 2012. Among them, 398 participants received CHM adjunct therapy; 217 participants received CHM monotherapy; 396 participants received aspirin alone therapy, the addition of other antiplatelet drug to aspirin therapy, or no treatment. The age of participants ranged from 40 to 84 years old, and the treatment duration ranged from 2 weeks to 3 months. All of the studies were performed in a single center and conducted in China. These studies related to a wide range of conditions including cardiovascular disease [33, 37, 38, 40, 41, 43, 46–48], cerebral infarction [34, 39, 45, 67], cardiocerebrovascular disease [35, 49], and high risk hypertension [36]. In 13 studies [33–37, 39, 41, 43, 45, 46, 48, 49, 67], the diagnostic criteria of AR are an average platelet aggregation rate ≥70% with 10 *μ*mol/L ADP as an agonist and/or ≥20% induced by 0.5 g/L AA [68]. Two studies adopted a maximum platelet aggregation rate ≥30% induced by 0.5 g/L AA as the diagnostic criteria of AR [38, 47]. Only 1 study [40] adopted a platelet aggregation ≥18 ohm with collage or ≥13 ohm with ADP as the diagnostic criteria of AR [69]. Five studies were three-group designed study [33, 37, 39–41]. In 2 studies, both CHM monotherapy and CHM adjunct therapy were compared with the regular aspirin treatment [37, 40]. Both CHM monotherapy and CHM adjunct therapy was compared with no treatment in 1 study [33]. In two studies, CHM adjunct therapy were compared with regular aspirin treatment and the addition of other antiplatelet drug [39, 41]. There was positive control in 5 studies. Among them, 3 studies used dipyridamole [34, 45, 67]; 1 study used clopidogrel [41]; 1 study used cilostazol [39]. All studies used platelet aggregation as primary outcome, and other indexes related to platelet aggregation were also used as outcome in 7 studies [34, 36, 38, 39, 41, 48, 49]. Clinical effective rate was observed in 4 studies [35, 47, 49, 67], but the standard was not completely the same in each study. Adverse events were reported in 7 studies [35, 36, 38, 40, 47, 49, 67], and the incidence of adverse event in treatment group was lower than in control group. The detailed characteristics of included studies are summarized in Table 1.

### 3.3. Description of the CHM and Its Prescription

Seven Chinese herbal prescriptions were tested in 12 (72.5%) of the 16 included studies, while the other 4 studies used single herb or active ingredients (Table 2). Tongxinluo capsule (TXLC) [39–41, 47] and Danshen-based prescriptions, including compound danshen dripping pill [36, 37], Qishenyiqi dripping pill [38], and Fufang Danshen injection [46], were the most frequently used standardized prescriptions. Danshen (danshen root; Radix Salviae Miltiorrhizae; *Salvia miltiorrhiza* Bge.), Huangqi (milkvetch root; Radix Astragali seu Hedysari; *Astragalus membranaceus* (Fisch.) Bge. var. Mongholicus (Bge.) Hsiao or *Astragalus membranaceus* (Fisch.) Bge.), Shuizhi (Leech; Hirudo; *Whitmania pigra* Whitman or *Hirudo nipponica* Whitman or *Whitmania acranulata* Whitman), and Jiangxiang (Rosewood; Lignum Dalbergiae Odoriferae; *Dalbergia odorifera* T. Chen) were the most frequently used single herbs, which were used for more than 3 times in all the trials. Nine of 11 preparations were Chinese patent medicine and have a rigorous quality control of herbal preparations, whereas the other 2 preparations were homemade [43, 49] (Table 2).

### 3.4. Risk of Bias in Included Studies

The methodological quality of RCTs was assessed independently using 12-item criteria from the Cochrane Back Review Group [66]. Risk of bias in included studies varied from 2/12 to 6/12, of average 3.7. Only 4 articles [35, 38, 40, 67] reported the method of random sequences generation, although all trials claimed that they were RCTs. Only 1 trial [38] reported allocation concealment, and none of the included studies mentioned blinding. The dropout data were not reported in 5 trials [35, 38, 40, 47, 67]. All the included studies have relatively small sample sizes and do not have formal sample size calculation. The methodological quality of included studies was summarized in Table 3.

### 3.5. Results of Individual Studies

Meta-analysis could not be performed owing to the high clinical heterogeneity and low methodological quality of the included studies. Additionally, the number of trials is too small to draw a meaningful funnel plot. Therefore, we also did not conduct the funnel plot analysis.

### 3.6. CHM Plus Aspirin versus No Treatment

One RCT [33] reported significant effects of CHM plus aspirin for reducing platelet aggregation rate compared with no treatment (*P* < 0.05). Although platelet aggregation rate was partly reduced after CHM monotherapy, there was no significant difference between CHM monotherapy group and no treatment control group (*P* > 0.05), Table 1.

### 3.7. CHM Monotherapy versus Aspirin 100 mg/d

There are 2 RCTs comparing CHM monotherapy with aspirin 100 mg/d therapy. Yin et al. [40] reported significant effect of CHM for reducing platelet aggregation rate when compared with aspirin 100 mg/d (*P* < 0.05). Chai et al. [37] showed that CHM has significant effect in reducing platelet aggregation induced by ADP (*P* < 0.05) but has no significant effect in reducing platelet aggregation induced by AA (*P* > 0.05) (Table 1).

### 3.8. CHM Monotherapy versus Aspirin 300 mg/d

There are 2 RCTs comparing CHM monotherapy with high dose aspirin therapy group (aspirin 300 mg/d) [35, 49]. Studies of Luo et al. [49] and Su [35] showed significant difference in the reduction of platelet aggregation rate when CHM monotherapy was compared with high dose aspirin therapy (*P* < 0.05). Based on the testing of TXB2 and TXB2/6-K-PGF 1 *α*, Su [35] indicated the significant effects of CHM for reducing platelet aggregation (*P* < 0.05). However, Luo et al. [49] indicated that AR patients receiving Zhilong Huoxue Tongyu capsule therapy have no significant difference in comparison with high dose aspirin therapy.

### 3.9. CHM Plus Aspirin versus Aspirin

There were 6 RCTs comparing CHM plus aspirin with aspirin 100 mg/d therapy. All 6 trials indicated more significant effect of CHM plus aspirin for reducing platelet aggregation rate compared with aspirin 100 mg/d therapy (*P* < 0.05) [36, 37, 39–41, 43].

### 3.10. CHM Plus Aspirin versus Dipyridamole or Cilostazol or Clopidogrel Plus Aspirin

There were 5 RCTs that compared CHM plus aspirin therapy with dipyridamole or cilostazol or clopidogrel plus aspirin therapy [34, 39, 41, 45, 67]. Three RCTs [34, 45, 67] showed significant difference in reduction of platelet aggregation rate when comparing CHM plus aspirin therapy with dipyridamole plus aspirin therapy (*P* < 0.05, *P* < 0.01). However, Song et al. [41] and Zhang et al. [39] indicated that AR patients receiving CHM plus aspirin therapy have no significant difference compared with cilostazol or clopidogrel plus aspirin therapy based on the rate of platelet aggregation and TX B2 production (*P* > 0.05).

### 3.11. CHM Plus Aspirin versus CHM

There were 7 RCTs which compared CHM plus aspirin therapy with CHM monotherapy [33, 37, 38, 40, 46–48] (Table 1). Six RCTs demonstrated that CHM plus aspirin therapy has significant effect on reducing platelet aggregation rate when compared with CHM monotherapy (*P* < 0.05, *P* < 0.01) [33, 37, 38, 46–48], whereas 1 study [40] showed no significant difference in platelet aggregation rate (*P* > 0.05).

### 3.12. Clinical Effective Rate

Clinical effective rate was reported in 4 out of 16 included studies [35, 47, 49, 67]. One RCT [47] indicated more significant effect of CHM plus aspirin for improving clinical effective rate compared with CHM monotherapy (*P* < 0.05). Liu et al. [67] reported significant difference in improving clinical effective rate when comparing CHM plus aspirin therapy with dipyridamole plus aspirin therapy (*P* < 0.05). Su [35] and Luo et al. [49] reported better effect of CHM for improving clinical effective rate of AR compared with high dose aspirin (*P* < 0.05).

### 3.13. Adverse Events

Among the 16 included studies, adverse events were reported in 7 studies [35, 36, 38, 40, 47, 49, 67]. There were no adverse events reported in 3 studies [38, 40, 47], while the left 4 studies reported the occurrence of adverse events, and statistical analysis was done. Guo et al. [36] reported adverse events such as acute myocardial infarction, cerebral infarction, or intracerebral hemorrhage, and the results showed that CHM was less likely to have adverse events (*P* < 0.05 or *P* < 0.01). A number of mild adverse events were observed in 3 studies [35, 49, 67], such as headache, dizziness, facial flushing, epigastric discomfort, and nausea, which can recover with no treatment. Liu et al. [67] reported 4 cases of adverse events in *Gingko biloba* plus aspirin group and 17 cases in dipyridamole plus aspirin group (*P* < 0.01). Su [35] reported 2 cases of adverse events in Diao Xinxuekang group and 16 cases in high dose aspirin group (*P* < 0.01). Luo et al. [49] reported 2 cases of adverse events in Zhilong Huowue Tongyu capsule group and 12 cases in high dose aspirin group (*P* < 0.01); see Table 1.

## 4. Discussion

### 4.1. Summary of Evidence

To our knowledge, this study is the first systematic review to determine the efficacy and safety of CHM for AR patients. A total of 1011 subjects in the 16 included trials of CHM for AR have emerged between 2006 and 2012, suggesting that the interests of the public and the medical profession in the use of CHM for AR have grown considerably in the recent years. Most of herbal preparations were Chinese patent medicine, which have a rigorous quality control. The current evidence is insufficient to recommend the routine use of CHMs for AR because of the poor methodological quality and lack of safety data.

### 4.2. Limitations

Several methodological limitations in the primary studies should be addressed. First, although all the included trials claimed to be RCTs, only 4 trials [35, 38, 40, 67] reported the method of random sequences generation. Lack of random description would lead to the possibility of bias. Only 1 trial [38] reported allocation concealment. Inadequate or unclear allocation concealment in trials could lead to an average 18% more “beneficial” than effect estimates from trials with adequate concealment (95% CI 5% to 29%) [70]. None of the included studies mentioned blinding, which may result in performance bias and detection bias. In addition, placebo control was not used in all included trials, which will make positive result in intervention group. Second, ITT analysis provides the least bias for a clinical trial [71]. CONSORT guidelines emphasized the necessity of ITT analysis on the reporting of randomized controlled trials [72]. However, there was no trial that mentioned analyzing data based on the ITT principle. Moreover, only 1 trial [36] mentioned 49 cases of withdrawal in the course of intervention. Third, selective bias is also a methodological limitation for this review. It is unclear if there was selective reporting in all included trials. High proportions of positive results were usually published in some Asian countries including China [73]. In this review, all included trials were from China. Therefore, we cannot exclude the possibility of selective bias. Fourth, members of the International Committee of Medical Journal Editors stated that all clinical trials must be registered in order to be publicated in September 2004 [74]. However, none of the included trials have been registered, which would affect the validity of trials. Our search strategy should have identified the majority of the available literature. However, we acknowledge the possibility that the review may not be fully comprehensive. Publication bias may also exist when nonsignificant findings remain unpublished, and thereby inflating the apparent magnitude of the effect. Fifth, an adequate sample size is vital to the design of randomized controlled trials [75]. All included studies have relatively small sample sizes and do not have formal sample size calculation, which would make this review's validity doubtable. Sixth, 12 different CHMs with great variation in terms of composition, dosage, and duration of interventions were reported in 16 included trials. There was a wide heterogeneity in the CHMs among the included studies because TCMs are composed of more than one herb and produce different therapeutic effects with different concentration proportions of the constituents [76]. This makes it difficult to recommend any specific CHM for clinical use.

## 5. Concluding Remarks

### 5.1. Implications for Practice

The available evidence from present systematic review is insufficient to recommend the routine use of CHM for AR because of the poor methodological quality, high clinical heterogeneity, and lack of safety data. However, CHMs appeared to be well tolerated in all included studies. Therefore, the efficacy and safety of CHM therapy for AR remain to be further determined. However, it should be remembered that a lack of scientific evidence does not necessarily mean that the treatment is ineffective [77]. TXLC [39–41, 47] and Danshen-based prescriptions, including compound danshen dripping pill [36, 37], Qishenyiqi dripping pill [38], and Fufang Danshen injection [46], were the most frequently used standardized Chinese patent medicine. These prescriptions have potentially clinical efficiency and their preparations have a rigorous quality control, which should be the priority for clinical use of AR. In addition, Danshen (danshen root), Huangqi (milkvetch root), Shuizhi (Leech), and Jiangxiang (Rosewood) as the most frequently used single herbs may contribute in composing a fundamental prescription for AR.

### 5.2. Implications for Research

CHMs are widely used in the treatment of AR. There are too many unanswered questions, making it impossible to draw positive or negative conclusions. The most frequently used standardized Chinese patent medicine such as TXLC and Danshen-based prescriptions as well as the most frequently used herbs, including danshen root, milkvetch root, Leech, and Rosewood that may contribute in composing a fundamental prescription, could be promising candidates for further clinical trials of AR. Owing to serious attention to methodological quality, we recommend that the CONSORT 2010 statement [78], RCTs investigating CHM [79], and CONSORT for TCM [80], which consist of a checklist to determine study quality and rigor, respectively, should be used as a guideline for further designing and reporting of RCTs.

The safety of herbal medicines has become a major concern to both national health authorities and the general public. Thus, World Health Organization (WHO) published WHO guidelines on safety monitoring of herbal medicines in pharmacovigilance systems in 2004 [81]. However, the risks caused by drug-herb interactions, especially for those involving anticoagulant/antiplatelet drugs and CHM, are often ignored or underestimated. A recent review of potential harmful interactions between anticoagulant/antiplatelet agents and CHM indicated that the additive anticoagulant or antiplatelet effects of the CHMs, especially danshen root, Danggui (Chinese angelica; Radix Angelicae Sinensis; *Angelica sinensis* (Oliv.) Diels), Jiang (ginger; Rhizoma Zingiberis Recens; *Zingiber officinale* Rosc.), Yinxingye (ginkgo leaf; Folium Ginkgo; *Ginkgo biloba* L.), Gancao (liquorice root; Radix Glycyrrhizae; *Glycyrrhiza uralensis* Fisch. or *Glycyrrhiza inflata* Bat. or *Glycyrrhiza glabra* L.), and Jianghuang (Turmeric; Rhizoma Curcumae Longae; *Curcuma longa* L.), could increase bleeding risks in those patients with cardio/cerebrovascular diseases [82]. However, adverse events in the present study were reported only in 7 studies [35, 36, 38, 40, 47, 49, 67]. Therefore, special attention should be paid to antiplatelet drug-CHMs interactions due to the potential risks of increased bleeding. Furthermore, many reports on adverse drug reactions demonstrated that the toxic effects with the use of CHM could be associated with hepatotoxicity, nephrotoxicity, nervous system damage, and so forth [83]. Therefore, adverse events should have been reported in each included trial in order to guide the clinical use of CHM for AR. A standard reporting format for ADR that has recently been developed should be used as a guideline of reporting adverse events and ADRs in the future clinical study [84].

## 6. Conclusions

Sixteen RCTs were identified in this systematic review for evaluating the efficacy and safety of various CHMs in the treatment of AR. Despite the apparent reported positive findings, it is premature to determine the efficacy and safety of CHM for the treatment of AR due to poor methodological quality and insufficient safety data. However, CHMs appeared to be well tolerated in all included studies. Therefore, CHM as a promising candidate are worthy of improvement and development for further clinical AR trials. Large sample-size and well-designed rigorous RCTs are needed to accurately determine the benefits and risks of CHM for AR.

## Figures and Tables

**Table 1:** Basic characteristics of the included studies.

Included trials	Type of disease (*n*)	Eligibility criteria of AR	Study designs	Interventions		Sample and characteristics (male/female; age)		Outcome index	Intergroup differences
				Trial	Control	Trial	Control		
Peng et al. 2011 [33]	Coronary heart disease (37)	AA# and ADP#	RCT (method unreported) and controlled nonblinded three-group design study	Huoxue capsule 12 pieces tid for 3 month	No treatment	12 —	13 —	Platelet aggregation rate	*P* > 0.05
				Huoxue capsule 12 pieces tid + aspirin 100 mg qd for 3 month	Huoxue capsule	12 —	12	Platelet aggregation rate	*P* < 0.05
				Huoxue capsule + aspirin	No treatment			Platelet aggregation rate	*P* < 0.05

Ma et al. 2012 [34]	Acute cerebral infarction (80)	AA# and ADP#	RCT (method unreported) and controlled nonblinded parallel study	Sodium ferulate100 mg, tid + aspirin 100 mg qd for 4 weeks	Aspirin 100 mg qd + dipyridamole 150 mg qd for 4 weeks	40 (M: 23, F: 17) Mean age: 63 y	40 (M: 21, F: 19) Mean age: 65 y	(1) Platelet aggregation rate (2) TXB2	(1) *P* < 0.05 (2) *P* < 0.05

Su 2012 [35]	Cardio/ Cerebrovascular disease (60)	AA# and ADP#	RCT (random number table) and controlled nonblinded parallel study	Diao Xin Xue kang 1.6 g, tid for 4 weeks	Aspirin 300 mg qd for 4 weeks	30 (M: 15, F: 15) Mean age: 62 y	30 (M: 13, F: 17) Mean age: 61.2 y	(1) Platelet aggregation rate (2) TXB2 (3) 6-K-PGF 1*α* (4) TXB2/6-K-PGF 1*α* (5) Clinical effective rate (6) Adverse events	(1) *P* < 0.05 (2) *P* < 0.05 (3) *P* < 0.05 (4) *P* < 0.05 (5) *P* < 0.05 (6) *P* < 0.01

Guo 2012 [36]	High risk hypertensive patients (103)	ADP#	RCT (method unreported) and controlled nonblinded parallel study	CDDP 270 mg tid + aspirin 100 mg qd for 1 month	Aspirin 100 mg qd for 1 month	50 —	53 —	(1) Platelet aggregation rate (2) Acute myocardial infarction (3) Cerebral infarction (4) Bleeding events	(1) *P* < 0.01 (2) *P* < 0.05 (3) *P* < 0.05 (4) *P* > 0.05

Chai et al. 2008 [37]	Cardiovascular disease (30)	AA# and ADP#	RCT (method unreported) and controlled nonblinded three-group design study	CDDP 10 pieces tid for 2 weeks	Aspirin 100 mg qd for 2 weeks	10 (M: 3, F: 7) Mean age: 67.70 ± 12.791 y	10 (M: 4, F: 6) Mean age: 12.31 ± 17.61 y	(1) ADP-induced platelet aggregation rate (2) AA-induced platelet aggregation rate	(1) *P* < 0.05 (2) *P* > 0.05
				CDDP 10 pieces tid + aspirin 100 mg qd for 2 weeks	CDDP	10 (M: 3, F: 7) Mean age: 67.4 ± 14.7 y		Platelet aggregation rate	*P* < 0.05 or *P* < 0.01
				CDDP + aspirin	Aspirin			Platelet aggregation rate	*P* < 0.01

Chen et al. 2008 [38]	Coronary heart disease (34)	AA#	RCT (random number table) and controlled nonblinded parallel study	Qishenyiqi pill 0.5 g tid + aspirin 100 mg qd for 4 weeks	Qishenyiqi Pill 0.5 g tid for 4 weeks	17 (M: 12, F: 22) Mean age: 53.4 ± 9.2 y	17	(1) Platelet aggregation rate (2) TG, VLDL (3) Adverse events	(1) *P* < 0.05 (2) *P* > 0.05 (3) *P* > 0.05

Zhang et al. 2010 [39]	Cerebral infarction (60)	ADP#	RCT (random number table) and controlled nonblinded three-group design study	TXLC 4 pieces tid + aspirin 100 mg qd for 1 month	Aspirin 100 mg qd for 1 month	20 (M: 20, F: 40) —	20 —	(1) Platelet aggregation rate (2) TXB2	(1) *P* < 0.05 (2) *P* < 0.05
				TXLC + Aspirin	Cilostazol 100 mg qd + aspirin 100 mg qd for 1 month		20 —	(1) Platelet aggregation rate (2) TXB2	(1) *P* > 0.05 (2) *P* > 0.05
				Cilostazol + aspirin	Aspirin			(1) Platelet aggregation rate (2) TXB2	(1) *P* < 0.01 (2) *P* < 0.01

Yin et al. 2010 [40]	Coronary heart disease (89)	ADP# and COL#	RCT (random number table) and controlled nonblinded three-group design study	TXLC 3 pieces tid for 1 month	Aspirin 100 mg qd for 1 month	30 (M: 19, F: 11) Mean age: 66.69 ± 10.56 y	29 (M: 17, F: 12) Mean age: 66.93 ± 10.75 y	(1) Platelet aggregation Rate (2) Adverse events	(1) *P* < 0.05 (2) *P* > 0.05
				TXLC 1 piece tid + aspirin 100 mg qd for 1 month	Aspirin	30 (M: 3, F: 7) Mean age: 67.4 ± 14.7 y		(1) Platelet aggregation rate (2) Adverse events	(1) *P* < 0.05 (2) *P* > 0.05
				TXLC + aspirin	TXLC			(1) Platelet aggregation rate (2) Adverse events	(1) *P* > 0.05 (2) *P* > 0.05

Song et al. 2008 [41]	Acute coronary syndrome (70)	AA# and ADP#	RCT (method unreported) and controlled nonblinded three-group design study	TXLC 4 pieces tid + aspirin 100 mg qd for 1 month	Aspirin 100 mg qd for 1 month	24 (M: 20, F: 50)	23	(1) Platelet aggregation rate (2) TXB2 (3) CRP	(1) *P* < 0.05 (2) *P* < 0.05 (3) *P* < 0.05
				TXLC + aspirin	Clopidogrel 75 mg qd + aspirin 100 mg qd for 1 month		23 —	(1) Platelet aggregation rate (2) TXB2 (3) CRP	(1) *P* > 0.05 (2) *P* > 0.05 (3) *P* > 0.05
				Clopidogrel + aspirin	Aspirin			(1) Platelet aggregation rate (2) TXB2 (3) CRP	(1) *P* < 0.05 (2) *P* < 0.01 (3) *P* < 0.05

Wu 2012 [43]	Cardiovascular disease (60)	AA# and ADP#	RCT (method unreported) and controlled nonblinded parallel study	Xuefuzhuyutang 1 dose/d + aspirin 100 mg qd for 4 weeks	Aspirin 100 mg qd for 4 weeks	30 (M: 38, F: 22) Mean age: 35–80 y	30	Platelet aggregation rate	*P* < 0.05

Liu 2010 [67]	Ischemic stroke (72)	AA# or ADP#	RCT (method unreported) and controlled nonblinded parallel study	*Gingko biloba* 2 pieces, tid + aspirin 100 mg qd for 1 month	Dipyridamole 150 mg qd + aspirin 100 mg qd for 1 month	36 (M: 21, F: 15) Mean age: 65 y	36 (M: 18, F: 18) Mean age: 67 y	(1) Platelet aggregation rate (2) Clinical effective rate (3) Adverse events	(1) *P* < 0.01 (2) *P* < 0.05 (3) *P* < 0.01

Liu 2008 [45]	Cerebral infarction (80)	ADP#	RCT (method unreported) and controlled nonblinded parallel study	Zhuyu Tongmai capsule 2 pieces tid + aspirin 100 mg qd for 1 month	Dipyridamole 150 mg qd + aspirin 100 mg qd for 1 month	40 (M: 21, F: 19)Mean age: 38–72 y	40 (M: 23, F: 17) Mean age: 41–75 y	Platelet aggregation rate	*P* < 0.01

Cheng et al. 2010 [46]	Chronic coronary disease (52)	AA# and ADP#	RCT (method unreported) and controlled nonblinded parallel study	Fufang Danshen injection 20 ml + aspirin 100 mg qd for 2 weeks	Fufang Danshen injection 20 ml for 2 weeks	26 (M: 14, F: 12) Mean age: 65 ± 9 y	26 (M: 14, F: 12) Mean age: 66 ± 8 y	Platelet aggregation rate	*P* < 0.05

Liu et al. 2006 [47]	Coronary heart disease (64)	AA# > 30%	RCT (method unreported) and controlled nonblinded parallel study	TXLC 4 pieces tid + aspirin 100 mg qd for 3 weeks	TXLC 4 pieces tid for 3 weeks	32 (M: 44, F: 20) Mean age: 69.4 ± 11.2 y	32	(1) Platelet aggregation rate (2) Clinical effective rate (3) Adverse events	(1) *P* < 0.05 (2) *P* < 0.05 (3) *P* > 0.05

Sun et al. 2009 [48]	Coronary heart disease (60)	AA# and ADP#	RCT (method unreported) and controlled nonblinded parallel study	Lumbrokinase enteric-coated capsules 60 wan IU tid + aspirin 100 mg qd for 1 month	Lumbrokinase enteric-coated capsules 60 wan IU tid for 1 month	30 (M: 16, F: 14) Mean age: 68.1 ± 14.7 y	30 (M: 13, F: 17) Mean age: 62.31 ± 17.61 y	Platelet aggregation rate	*P* < 0.01

Luo et al. 2012 [49]	Cardio/ Cerebrovascular disease (60)	AA# and ADP#	RCT (method unreported) and controlled nonblinded parallel study	Zhilonghuoxuetongyu capsule 1.6 g, tid for 4 weeks	Aspirin 300 mg qd for 4 weeks	30 (M: 15, F: 15) Mean age: 43–70 y	30 (M: 13, F: 17) Mean age: 41–70 y	(1) Platelet aggregation rate (2) TXB2 (3) 6-K-PGF 1*α* (4) TXB2/6-K-PGF 1*α* (5) Clinical effective rate (6) Adverse events	(1) *P* < 0.05 (2) *P* > 0.05 (3) *P* < 0.05 (4) *P* > 0.05 (5) *P* < 0.05 (6) *P* < 0.01

AA#: arachidonic acid induced platelet aggregation rate > 20%; ADP#: adenosine diphosphate induced platelet aggregation rate > 70%; COL#: collagen induced platelet aggregation rate > 30%; CDDP: compound Danshen dripping pill; TXB2: thromboxane B2; 6-K-PGF 1*α*: 6-keto-prostaglandin F1a; TG: triglyceride; VLDL: very-low-density lipoprotein; CRP: C response protein; TXLC: Tongxinluo capsule.

**Table 2:** Chinese herbal prescription or single herb or active ingredients for aspirin resistance in the 16 reviewed studies.

Reference	Chinese herbal prescription or single herb or active ingredients	Content (Chinese pinyin, English herb name, Latin herb name, Family)	Preparations/dosage	Chinese patent medicine
Zhang et al. 2010 [39], Yin et al. 2010 [40], Song et al. 2008 [41], Liu et al. 2006 [47]	Tongxinluo capsule	Renshen (Ginseng; Radix Ginseng; *Panax ginseng* C. A. Mey.), Shuizhi (Leech; Hirudo; *Whitmania pigra* Whitman or *Hirudo nipponica* Whitman or *Whitmania acranulata* Whitman), Quanxie (Scorpion; Scorpio; *Buthus martensii* Karsch), Chishao (peony root; Radix Paeoniae Rubra; *Paeonia lactiflora* Pall. or *Paeonia veitchii* Lynch), Chantui (cicada slough; Periostracum Cicadae; *Cryptotympana pustulata* Fabricius), Tubiechong (ground beetle; Eupolyphaga Seu Steleophaga; *Eupolyphaga sinensis* Walker or *Steleophaga plancyi* (Boleny)), Wusong (Centipede; Scolopendra; *Scolopendra subspinipes mutilans* L. Koch), Tanxiang (Sandalwood; Lignum Santali Albi; *Santalum album* L.), Jiangxiang (Rosewood; Lignum Dalbergiae Odoriferae; *Dalbergia odorifera* T. Chen), Ruxiang (Frankincense; Olibanum; *Boswellia carterii* Birdw. or *Boswellia bhaw-dajiana* Birdw or *Boswellia neglecta* M. Moore), Suanzaoren (spine date seed; Semen Ziziphi Spinosae; *Ziziphus jujuba* Mill. var. or *spinosa* (Bunge) Hu ex H. F. Chou), Bingpian (Borneol; Borneolum Syntheticum)	Capsule/3 or 4 capsules tid	Yes

Chai et al. 2008 [37], Guo 2012 [36]	Compound danshen dripping pill	Danshen (danshen root; Radix *Salviae Miltiorrhizae*; Salvia miltiorrhiza Bge)., Sanqi (Sanqi; Radix Notoginseng; *Panax notoginseng* (Burk.) F. H. Chen), Bingpian (Borneol; Borneolum Syntheticum)	Dripping pill/10 pills tid	Yes

Wu 2012 [43]	Xuefu Zhuyu Decoction	Danggui (Chinese angelica; Radix Angelicae Sinensis; *Angelica sinensis* (Oliv.) Diels) 9 g, Shengdihuang (unprocessed rehmannia root; Radix Rehmanniae Recens) 9 g, Taoren (peach seed; Semen Persicae; *Amygdalus persica* L. or *Amygdalus davidiana* (Carr.) C. de Vos ex Henry) 12 g, Honghua (Safflower; Flos Carthami; *Carthamus tinctorius* L.) 9 g, Zhike (orange fruit; Fructus Aurantii; *Citrus aurantium* L.) 6 g, Chishao (peony root; Radix Paeoniae Rubra; *Paeonia lactiflora* Pall. or *Paeonia veitchii* Lynch) 6 g, Chuanxiong (sichuan lovage rhizome; Rhizoma Ligustici Chuanxiong; *Ligusticum chuanxiong* Hort.) 5 g, Chaihu (Chinese thorowax root; Radix Bupleuri; *Bupleurum chinensis* DC. or *Bupleurum scorzonerifolium* Willd.) 3 g, Jiegeng (platycodon root; Radix Platycodonis; *Platycodon grandiflorus* (Jacq.) A. DC.) 5 g, Niuxi (two-toothed achyranthes root; Radix Achyranthis Bidentatae; *Achyranthes bidentata* Bl.) 9 g, Gancao (liquorice root; Radix Glycyrrhizae; *Glycyrrhiza uralensis* Fisch. or *Glycyrrhiza inflata* Bat. or *Glycyrrhiza glabra* L.) 3 g.	Decoction//1 dose qd	No

Peng et al. 2011 [33]	Huoxue capsule	Xuefu Zhuyu decoction minor Jiegeng (platycodon root; Radix Platycodonis; *Platycodon grandiflorus* (Jacq.) A. DC.), plus Huangqi (milkvetch root; Radix Astragali seu Hedysari; *Astragalus membranaceus* (Fisch.) Bge. var. Mongholicus (Bge.) Hsiao or *Astragalus membranaceus* (Fisch.) Bge.), and Suanzaoren (spine date seed; Semen Ziziphi Spinosae; *Ziziphus jujuba* Mill. var. or *spinosa* (Bunge) Hu ex H. F. Chou)	Capsule/2 capsules tid	Yes

Chen et al. 2008 [38]	Qisheyiqi dripping pill	Huangqi (milkvetch root; Radix Astragali seu Hedysari; *Astragalus membranaceus* (Fisch.) Bge. var. Mongholicus (Bge.) Hsiao or *Astragalus membranaceus* (Fisch.) Bge.), Danshen (danshen root; Radix Salviae Miltiorrhizae; *Salvia miltiorrhiza* Bge.), Sanqi (Sanqi; Radix Notoginseng; *Panax notoginseng* (Burk.) F. H. Chen), oil of Jiangxiang (Rosewood; Lignum Dalbergiae Odoriferae; *Dallbergia odarifera* T. Chen)	Dripping pill/0.5 g bid	Yes

Liu 2008 [45]	Zhuoyu Tongmai capsule	Mengchong (Tabanus; Gadfly), Shuizhi (Leech; Hirudo; *Whitmania pigra* Whitman or *Hirudo nipponica* Whitman or *Whitmania acranulata* Whitman), Taoren (peach seed; Semen Persicae; *Amygdalus persica* L. or *Amygdalus davidiana* (Carr.) C. de Vos ex Henry), Dahuang (rhubarb root and rhizome; Radix et Rhizoma Rhei; *Rheum palmatum* L. or *Rheum tanguticum* Maxim. ex Balf. or *Rheum officinale* Baill.)	Capsule/2 capsules tid	Yes

Cheng et al. 2010 [46]	Fufang Danshen injection	Danshen (danshen root; Radix Salviae Miltiorrhizae; *Salvia miltiorrhiza* Bge.), Jiangxiang (Rosewood; Lignum Dalbergiae Odoriferae; *Dalbergia odorifera* T. Chen)	Injection/20 ml qd	Yes

Luo et al. 2012 [49]	Zhilong Huoxue Tongyu capsule	Huangqi (milkvetch root; Radix Astragali seu Hedysari; *Astragalus membranaceus* (Fisch.) Bge. var. Mongolicus (Bge.) Hsiao or *Astragalus membranaceus* (Fisch.) Bge.), Guizhi (cassia twig; Ramulus Cinnamomi; *Cinnamomum cassia* Presl), Daxueteng (sargentgloryvine stem; Caulis Sargentodoxae; *Sargentodoxa cuneata* (Oliv.) Rehd. et Wils.), Shuizhi (Leech; Hirudo; *Whitmania pigra* Whitman or *Hirudo nipponica* Whitman or *Whitmania acranulata* Whitman), Quanxie (Scorpion; Scorpio; *Buthus martensii* Karsch), Dilong (Earthworm; Lumbricus; *Pheretima aspergillum* (E. Perrier) or *Pheretima vulgaris* Chen or *Pheretima guillelmi* (Michaelsen) or *Pheretima pectinifera* Michaelsen )	Capsule/1.6 g tid	No

Ma et al. 2012 [34]	Sodium ferulate tablets	The sodium salt of ferulic acid. It is found in the root of *Angelica sinensis*. Ferulic acid can also be extracted from the root of the Chinese herb *Ligusticum chuanxiong*.	Tablets/100 mg tid	Yes

Su 2012 [35]	DiaoXinxuekang capsule	A dry extract of the root of *Dioscorea nipponica* Makino	Capsule/1.6 g tid	Yes

Liu 2010 [67]	*Gingko biloba* tablets	Extract of Yinxingye (ginkgo leaf; Folium Ginkgo; *Ginkgo biloba* L.)	Tablets/2 tablets tid	Yes

Sun et al. 2009 [48]	Lumbrokinase enteric-coated capsules	A group of proteolytic enzymes derived from the earthworm *Lumbricus rubellus *	Enteric-coated capsule/60,000 IU tid	Yes

**Table 3:** The methodological quality of included studies.

	A	B	C	D	E	F	G	H	I	J	K	L	Total +	Total −	Total ?
Peng et al. 2011 [33]	?	−	−	−	−	?	?	?	+	+	?	+	3	4	5
Ma et al. 2012 [34]	?	−	−	−	−	?	?	?	+	+	?	+	3	4	5
Su 2012 [35]	+	−	−	−	−	?	+	?	+	+	?	+	5	4	2
Guo 2012 [36]	?	−	−	−	−	?	−	?	+	+	?	+	3	5	3
Chai et al. 2008 [37]	?	−	−	−	−	?	?	?	−	+	?	+	2	5	5
Chen et al. 2008 [38]	+	+	−	−	−	?	+	?	+	+	?	+	6	4	3
Zhang et al. 2010 [39]	?	−	−	−	−	?	?	?	+	+	?	+	3	4	6
Yin et al. 2010 [40]	+	−	−	−	−	?	+	?	+	+	+	+	5	4	3
Song et al. 2008 [41]	?	−	−	−	−	?	?	?	+	+	?	+	3	4	5
Wu 2012 [43]	?	−	−	−	−	?	?	?	+	+	?	+	3	4	5
Liu 2010 [67]	+	−	−	−	−	?	+	?	+	+	?	+	5	4	2
Liu 2008 [45]	?	−	−	−	−	?	?	?	+	+	?	+	3	4	5
Cheng et al. 2010 [46]	?	−	−	−	−	?	?	?	+	+	?	+	3	4	5
Liu et al. 2006 [47]	?	−	−	−	−	?	+	?	+	+	?	+	4	4	4
Sun et al. 2009 [48]	?	−	−	−	−	?	?	?	+	+	?	+	3	4	5
Luo et al. 2012 [49]	?	−	−	−	−	?	?	?	+	+	?	+	3	4	5

A: adequate sequence generation; B: concealment of allocation; C: blinding (patient); D: blinding (investigator); E: blinding (assessor); F: incomplete outcome data addressed (ITT analysis); G: incomplete outcome data addressed (dropouts); H: free of selective reporting; I: similarity at baseline; J: cointerventions constant; K: compliance acceptable; L: similar timing outcome assessments. +: yes, −: no, and ?: unclear.
